# Weekend Texting Screen Time and Incident Nicotine Use in Early Adolescents: Longitudinal Analysis of the Adolescent Brain Cognitive Development Study

**DOI:** 10.2196/100303

**Published:** 2026-10-13

**Authors:** Gabriel Zamora, Michael L Thomas, Alejandro D Meruelo

**Affiliations:** 1 Department of Psychiatry, University of California San Diego, 9500 Gilman Dr, La Jolla, CA, 92093, United States, 1 619-543-5221; 2 Department of Psychology, Colorado State University, Fort Collins, CO, United States

**Keywords:** adolescent nicotine initiation, screen time, SMS text messaging, negative urgency, impulsivity, longitudinal study

## Abstract

**Background:**

Adolescent nicotine use initiation remains a significant public health concern. While screen-based behaviors have been linked to substance use, few prospective studies have examined specific forms of interactive screen engagement or tested potential psychological mechanisms underlying this association.

**Objective:**

This study aimed to examine whether weekend texting screen time was prospectively associated with subsequent nicotine use initiation among early adolescents and evaluate whether negative urgency statistically accounted for part of this association.

**Methods:**

We analyzed longitudinal data from a large cohort of adolescents who were nicotine naive at baseline. Weekend texting screen time (hours) was assessed via self-report. Negative urgency, reflecting impulsive action during negative affect, was measured using the UPPS (Urgency, Premeditation, Perseverance, and Sensation Seeking) impulsivity scale. Incident nicotine use initiation was modeled using Cox proportional hazards regression, adjusting for sex, age, race, ethnicity, and household income. We evaluated whether adjustment for negative urgency attenuated the texting-initiation association and conducted a sensitivity analysis restricted to participants for whom texting assessment, negative urgency assessment, and nicotine use initiation were temporally ordered.

**Results:**

Among 5,965 nicotine-naïve adolescents followed prospectively, 239 (4%) initiated nicotine use. Greater weekend texting was associated with increased hazard of nicotine use initiation (per hour: hazard ratio [HR]=1.07, 95% CI 1.04‐1.11). A 5-hour increase corresponded to an approximately 30% to 40% higher hazard of initiation. Weekend texting was positively associated with negative urgency (β=0.079; *P*<.001), and negative urgency independently predicted nicotine use initiation (per point: HR=1.19, 95% CI 1.13‐1.25). Inclusion of negative urgency slightly attenuated the texting association (per hour: HR=1.06, 95% CI 1.02‐1.09), although attenuation was modest. In standardized models, a 1-SD increase in weekend texting was associated with a 21% higher hazard of initiation (HR=1.21, 95% CI 1.10‐1.31). In a sensitivity analysis restricted to participants with temporally ordered assessments and nicotine use initiation, weekend texting remained associated with nicotine use initiation (HR=1.11, 95% CI 1.06‐1.16), and negative urgency remained independently associated with use initiation (HR=1.11, 95% CI 1.02‐1.21). Adjustment for negative urgency attenuated the texting log-hazard coefficient by 6.8%.

**Conclusions:**

Greater weekend texting screen time was prospectively associated with subsequent nicotine use initiation among early adolescents. Negative urgency was independently associated with nicotine use initiation and modestly attenuated the texting-initiation association. These findings are consistent with overlapping contributions of digital engagement and affect-driven impulsivity to early nicotine risk but do not establish a causal mediating pathway.

## Introduction

Adolescent nicotine use initiation remains a major public health concern as early onset of use is associated with increased risk of nicotine dependence, polysubstance use, and long-term adverse health outcomes. Although traditional combustible cigarette use has declined in recent years, the proliferation of electronic nicotine delivery systems and related products has sustained elevated rates of experimentation and initiation during adolescence [1,2]. Longitudinal studies indicate that early e-cigarette use is associated with increased likelihood of subsequent combustible cigarette use initiation [3,4]. Identifying modifiable behavioral risk factors that precede nicotine use onset is therefore critical for prevention efforts, particularly given that adolescence is a sensitive developmental period characterized by increased susceptibility to social influence and risk behavior [5].

Screen-based technology use has increased dramatically among youth over the past decade. Adolescents now spend substantial time engaging in texting, messaging, and other forms of digital social interaction [6,7], particularly during weekends, when structured activities are reduced. Prior research has linked greater overall screen time with substance use, including alcohol and nicotine [8,9], but findings have been inconsistent and often limited by cross-sectional designs, aggregation of heterogeneous screen behaviors, or lack of temporal ordering [10]. Importantly, different forms and contexts of screen engagement may not carry equivalent information regarding substance use vulnerability. Texting and messaging are socially interactive behaviors that may capture peer communication and interpersonal engagement more directly than passive screen activities such as television viewing. We focused specifically on weekend texting because weekends are periods with fewer school-related constraints and, therefore, weekend use may more closely reflect discretionary patterns of peer-oriented digital engagement. This distinction does not imply that weekday texting or other interactive screen behaviors are unimportant; rather, weekend texting was examined as a specific, socially interactive exposure that may be particularly informative during periods of relatively unstructured time.

Impulsivity-related traits may represent one individual-level factor associated with both digital engagement and substance use risk. Negative urgency—the tendency to act impulsively during states of negative affect—is a well-established predictor of adolescent substance use initiation and escalation [11,12]. Although negative urgency is conceptualized as an impulsivity-related personality disposition, longitudinal research indicates that negative urgency itself shows meaningful developmental change across adolescence, increasing during early adolescence before leveling off [13]. Thus, negative urgency is not developmentally invariant during this period. Youth with elevated negative urgency are more likely to engage in maladaptive coping behaviors, including nicotine use, particularly in emotionally charged contexts [14]. Interactive digital environments may also involve emotionally salient social feedback, peer reinforcement, and interpersonal stressors [15,16]. Accordingly, greater engagement in these environments and negative urgency may jointly characterize adolescents at elevated risk of substance use initiation. However, whether negative urgency statistically accounts for part of the longitudinal association between digital engagement and subsequent nicotine use initiation remains unclear.

Despite this conceptual overlap, the extent to which impulsivity traits help explain the relationship between screen engagement and nicotine use initiation remains unclear. A key gap in the literature is the lack of longitudinal, prospective investigations testing whether specific forms of screen time are prospectively associated with incident nicotine use initiation among nicotine-naive adolescents and whether negative urgency statistically accounts for part of this longitudinal association. We conceptualized negative urgency as an affect-related impulsivity characteristic associated with vulnerability to risky behavior rather than as evidence that nicotine use initiation occurs during or immediately following texting episodes. Most prior studies have relied on cross-sectional designs, limiting causal inference, or have not examined mediation mechanisms grounded in personality and affect regulation frameworks [17,18]. Clarifying whether impulsivity partially explains this association has implications for both digital health guidance and targeted prevention strategies.

The present study addresses this gap using prospective data from a large cohort followed from early adolescence. We examined whether weekend texting screen time was associated with subsequent nicotine use initiation among youth who were nicotine naive at baseline. We further evaluated whether negative urgency was associated with both texting behavior and nicotine use initiation and whether adjustment for negative urgency attenuated the texting-initiation association.

We tested three primary hypotheses. First, greater weekend texting screen time would be associated with increased risk of incident nicotine use initiation. Second, higher texting screen time would be associated with greater negative urgency. Third, higher negative urgency would be independently associated with nicotine use initiation, and adjustment for negative urgency would partially attenuate the association between texting screen time and use initiation.

By focusing on a specific, socially interactive form of screen engagement and using prospective time-to-event analyses, this study aimed to clarify behavioral and personality mechanisms that may contribute to early nicotine use risk in contemporary digital environments.

## Methods

### Study Design and Participants

Data were drawn from a large longitudinal cohort of adolescents, the Adolescent Brain Cognitive Development (ABCD) study, assessed at multiple time points [19]. The assessments used in the present analyses took place between 2019 and 2024. Baseline Screen Time Questionnaire (STQ) assessments in the analytic sample were completed between March 2019 and October 2023, with subsequent UPPS (Urgency, Premeditation, Perseverance, and Sensation Seeking) and substance use follow-up assessments extending up to March 2024. The present analyses were restricted to participants with available STQ data [20] and UPPS impulsivity data [21], as well as information regarding nicotine use initiation [22]. To establish temporal ordering, participants who reported nicotine use initiation on or before the STQ assessment were excluded, yielding a nicotine-naive baseline cohort. Participants were further restricted to those with complete covariate data required for multivariable modeling. The final analytic sample consisted of adolescents followed prospectively for incident nicotine use initiation.

### Measures

#### Weekend Texting Screen Time

Weekend texting screen time at baseline was assessed using a self-report item from the STQ [20] that asked participants to report the number of hours spent texting or messaging on a cell phone, tablet, computer, or similar electronic device during weekends. Responses were recorded in hours. The distribution of texting time was right skewed, with many participants reporting low levels of exposure and a smaller subset reporting higher durations.

#### Negative Urgency

Impulsivity was assessed using the negative urgency subscale of the UPPS impulsive behavior measure [21]. Negative urgency reflects the tendency to act impulsively during states of negative affect. A summed score was used for analysis, with higher scores indicating greater urgency. The negative urgency score was treated as a continuous variable in all models. For each participant, negative urgency was obtained from the earliest available UPPS assessment taking place on or after the baseline STQ assessment. Eligible assessments were restricted to those taking place within 2500 days after the STQ assessment. In the primary analytic sample, 52.2% of selected UPPS assessments took place within 1 day of the STQ assessment; the median interval was less than 1 day (IQR <1‐321; range <1‐590 days).

#### Nicotine Use Initiation

Incident nicotine use initiation was defined as first reported use of nicotine products occurring after the baseline STQ assessment. Time to event was calculated from the date of the STQ assessment to the reported onset date of nicotine use. Participants who did not initiate nicotine use during follow-up were censored at their last available assessment.

### Covariates

All models were adjusted for demographic covariates, including sex, age at baseline assessment, race, ethnicity, and household income. Income was included as a continuous variable. Analyses were conducted using a complete-case approach, with participants missing covariate data excluded from adjusted models.

### Statistical Analysis

Descriptive statistics were calculated for weekend texting screen time and negative urgency. Weekend texting screen time was assessed at the baseline STQ assessment. For each participant, negative urgency was obtained from the earliest available UPPS assessment taking place on or after the STQ assessment within the specified 2500-day eligibility window. Because the timing of the selected UPPS assessment varied across participants, including assessments taking place on the same day as the STQ, negative urgency was not treated as a uniformly subsequent assessment. Associations between baseline texting screen time and follow-up negative urgency were evaluated using linear regression models adjusted for demographic covariates.

Prospective associations between baseline texting screen time and incident nicotine use initiation were examined using Cox proportional hazards regression models [23]. The primary model estimated the total association between baseline texting screen time and time to nicotine use initiation, adjusting for demographic covariates. Participants were restricted to those who were nicotine naive at baseline. Time to event was defined as the number of days from the baseline texting assessment to the first recorded nicotine use onset date derived from longitudinal substance use data. Participants without nicotine use initiation were censored at their last available follow-up assessment. Because Cox proportional hazards models explicitly incorporated each participant’s observed time-to-event or censoring time, follow-up duration was not included as an additional covariate. We nevertheless examined the association between weekend texting screen time and follow-up duration to assess potential differential observation by exposure. To enhance interpretability, hazard ratios (HRs) were reported per 1-hour increase in weekend texting screen time and were additionally expressed for increases corresponding to 1 SD, 1 IQR, and 5 hours.

To examine whether negative urgency statistically accounted for part of the association between texting screen time and nicotine use initiation, a second Cox model included both texting screen time and negative urgency. Change in the texting coefficient following inclusion of negative urgency was used to quantify attenuation of the association. Because negative urgency was not uniformly assessed after STQ and before nicotine use initiation, these analyses were interpreted as associational rather than as establishing causal mediation.

As a sensitivity analysis addressing temporal ordering, we repeated the Cox analyses after excluding participants whose recorded nicotine use initiation occurred before or on the date of their selected UPPS assessment. This restriction ensured the sequence STQ assessment→UPPS negative urgency assessment→nicotine use initiation among participants experiencing the outcome. The time origin remained the baseline STQ assessment to preserve comparability with the primary survival analysis. Models used the same demographic covariates and family-level clustering as the primary analyses.

An exploratory product-of-coefficients analysis was additionally conducted on the log-hazard scale with nonparametric bootstrap CIs. Because the temporal ordering required for causal mediation was not consistently present in the primary sample, this analysis was interpreted only as a statistical breakdown of associations and not as an estimate of a causal indirect effect or causal proportion mediated.

Cox models incorporated clustering at the family level to account for nonindependence of observations. Proportional hazards assumptions were evaluated using Schoenfeld residual tests [24]. No violations of the proportional hazards assumption were detected. All analyses were conducted in R (R Foundation for Statistical Computing) [25] using the *data.table* and *survival* packages [26,27], along with custom code for bootstrap-based mediation analyses. Statistical significance was evaluated at a 2-sided α level of .05.

### Ethical Considerations

The ABCD study protocol was approved by the centralized Institutional Review Board (IRB) at the University of California, San Diego (160091), with participating study sites obtaining or relying on appropriate local IRB approval. Parents or legal guardians provided written informed consent, and participating youth provided assent. The present study involved secondary analysis of deidentified ABCD study data obtained through the National Institute of Mental Health Data Archive and was determined to be exempt from review by the University of California, San Diego IRB (815599). All study procedures were conducted in accordance with applicable ethical guidelines and the principles of the Declaration of Helsinki.

## Results

### Sample Characteristics

Of 8048 participants with available STQ and UPPS data, 771 individuals who reported nicotine use initiation on or before the STQ assessment were excluded to ensure a nicotine-naive baseline cohort. This yielded 7277 eligible participants. After restricting to individuals with complete exposure and mediator data and valid follow-up time, 5965 participants were included in the primary Cox proportional hazards analyses, during which 239 incident nicotine use initiation events were observed (Table 1).

**Table 1.** Baseline characteristics of participants who did and did not initiate nicotine use during follow-up. *P* values reflect unadjusted comparisons between groups using *t* tests for continuous variables, chi-square tests for categorical variables, and Wilcoxon rank-sum tests for household income.

Characteristics	No nicotine use onset (n=5726)	Nicotine use onset (n=239)	*P* value
Age at STQ^a^ (years), mean (SD)	9.92 (0.62)	10.01 (0.61)	.02
Sex, n (%)			<.001
Male	3073 (53.7)	101 (42.3)	—^b^
Female	2653 (46.3)	138 (57.7)	—
Race, n/N (%)			.12
Asian	138/5712 (2.4)	2/239 (0.8)	—
Black	934/5712 (16.4)	33/239 (13.8)	—
Multiracial	612/5712 (10.7)	23/239 (9.6)	—
White	3735/5712 (65.4)	162/239 (67.8)	—
Other	293/5712 (5.1)	19/239 (7.9)	—
Ethnicity, n/N (%)			.13
Not Hispanic or Latino	4648/5647 (82.3)	186/237 (78.5)	—
Hispanic or Latino	999/5647 (17.7)	51/237 (21.5)	—
Household income category score, median (IQR)	8.0 (6.0-9.0)	8.0 (6.0-9.0)	.77
Weekend texting screen time (hours), mean (SD)	1.53 (2.67)	2.20 (2.97)	<.001
UPPS^c^ negative urgency (possible range 4-16), mean (SD)	8.36 (2.43)	9.41 (2.45)	<.001
Follow-up time (days), median (IQR)	314.7 (238.8-367.8)	193.5 (102.9-279.3)	<.001

a STQ: Screen Time Questionnaire.

b Not applicable.

c UPPS: Urgency, Premeditation, Perseverance, and Sensation Seeking.

In the primary analytic sample, participants had a median follow-up of 311 (IQR 232-366) days, with a maximum follow-up of 1407 days. A total of 28.7% (2827/9858) of participants had no follow-up Substance Use Interview assessment after the STQ, and 10.9% (1074/9858) had near-immediate follow-up (≤30 days). Follow-up duration was weakly inversely associated with weekend texting screen time (*r*=−0.044), indicating minimal differential follow-up by exposure (Table 2).

**Table 2.** Summary of follow-up time and observation density after Screen Time Questionnaire (STQ) baseline. Follow-up is defined relative to the baseline STQ assessment using Substance Use Interview (SUI) visit data. Post-STQ visits reflect the number of follow-up assessments per participant.

Characteristics	Values
Participants with baseline STQ, n	9858
No SUI follow-up after STQ, n/N (%)	2827/9858 (28.7)
Number of SUI visits after the STQ, n/N (%)	
0	2827/9858 (28.7)
1	5519/9858 (56.0)
2	1469/9858 (14.9)
≥3	43/9858 (0.4)
Follow-up time (days; after the STQ)	
Median (IQR)	307 (216‐364)
Mean (SD)	275 (143)
Maximum	1407
Near-immediate follow-up (≤30 days), n/N (%)	1074/9858 (10.9)
Correlation: exposure and follow-up, *r*	−0.044
Slope (days per 1 hour of screen time)	−2.23

Weekend texting screen time and UPPS negative urgency scores were approximately normally distributed after cleaning. The negative urgency score had a mean of 8.36 (SD 2.43).

### Association Between Weekend Texting Screen Time and Negative Urgency

In linear regression models, greater weekend texting screen time was significantly associated with higher negative urgency scores (β=0.079; *P*<.001; Tables 3 and 4). Each additional hour of weekend texting screen time was associated with a modest but statistically robust increase in negative urgency, although the overall variance explained was small (*R*^2^=0.008).

**Table 3.** Cox proportional hazards models estimating hazard ratios (HRs) for incident nicotine use^a^.

Predictor	Model 1 HR (95% CI)	*P* value	Model 2 HR (95% CI)	*P* value
Weekend texting screen time (exposure)	1.07 (1.04‐1.11)	<.001	1.06 (1.02‐1.09)	<.001
Negative urgency (mediator)	—^b^	—	1.19 (1.13‐1.25)	<.001

a Model 1 includes weekend texting screen time only; model 2 includes both weekend texting screen time and UPPS negative urgency. HRs represent the change in risk per 1-hour increase in weekend texting screen time and per 1-unit increase in negative urgency score.

b The predictor was not included in the model.

**Table 4.** Standardized effects per 1-SD increase in exposure and mediator variables, including standardized estimates from models before and after adjustment for negative urgency; the exploratory product-of-coefficients estimate is also shown.

Predictor	Standardized HR^a^ (95% CI)
Weekend texting screen time (primary model)	1.21 (1.10‐1.31)
Weekend texting screen time (adjusted for negative urgency)	1.17 (1.06‐1.28)
Negative urgency	1.49 (1.29‐1.73)
Exploratory product-of-coefficients estimate	1.04 (1.02‐1.06)

a HR: hazard ratio.

Models were adjusted for demographic covariates (sex, age, race, ethnicity, and household income); full parameter estimates are provided in Table S1 in Multimedia Appendix 1.

### Weekend Texting and Nicotine Use Initiation

In Cox proportional hazards models, greater weekend texting screen time was significantly associated with increased risk of incident nicotine use initiation. Each additional hour of weekend texting screen time was associated with a 7% higher hazard of initiation, corresponding to an HR of 1.07 with a 95% CI of 1.04 to 1.11. Model discrimination was modest, with a concordance of 0.617. There was no evidence of violation of the proportional hazards assumption.

The magnitude of this association was clinically meaningful when scaled to larger differences in exposure. A 5-hour increase in weekend texting screen time corresponded to approximately a 40% higher hazard of nicotine use initiation. When standardized, a 1-SD increase in weekend texting screen time was associated with a 21% higher hazard of nicotine use initiation (HR=1.21, 95% CI 1.10‐1.31).

### Association After Adjustment for Negative Urgency

When UPPS negative urgency was included in the model, it independently predicted nicotine use initiation, with each 1-point increase associated with a 19% higher hazard. The association between weekend texting screen time and nicotine use initiation was modestly reduced after adjustment for negative urgency (HR=1.06 per hour), although the magnitude of this attenuation was small. The persistence of the texting association after adjustment indicates that negative urgency statistically accounted for only a portion of the observed association. Because negative urgency was not uniformly assessed before nicotine use initiation, this attenuation should not be interpreted as evidence of a causal indirect effect. In standardized models, the adjusted for negative urgency effect of weekend texting screen time remained statistically significant (HR=1.17, 95% CI 1.06‐1.28), whereas a 1-SD increase in negative urgency was associated with a 49% higher hazard of nicotine use initiation (HR=1.49, 95% CI 1.29‐1.73).

### Exploratory Bootstrap Statistical Decomposition

Exploratory bootstrap analyses yielded a statistically significant product-of-coefficients estimate for the association involving weekend texting, negative urgency, and nicotine use initiation (Table 5). On the log-hazard scale, the product estimate was 0.014 (95% CI 0.008‐0.022). The standardized product estimate was 0.0365 (95% CI 0.0201‐0.0561). Because negative urgency did not uniformly precede nicotine initiation in the primary analytic sample, these estimates are presented as statistical decompositions rather than causal indirect effects, and the corresponding proportion should not be interpreted as the proportion of the total effect causally mediated through negative urgency.

**Table 5.** Bootstrap mediation results estimating indirect effects of weekend texting screen time on nicotine use initiation through UPPS negative urgency^a^.

Effect	Estimate (95% CI)
Indirect effect—log-hazard	0.014 (0.008-0.022)
Indirect effect—standardized log-hazard	0.0365 (0.0201-0.0561)
Standardized indirect HR^b^	1.04 (1.02-1.06)

a Effects are presented on both the log-hazard scale and as hazard ratios. Standardized estimates reflect effects per 1 SD increase in exposure. CIs are derived from bootstrap resampling.

b HR: hazard ratio.

### Sensitivity Analysis Restricting to Temporally Ordered Assessments

Because negative urgency did not uniformly precede nicotine use initiation in the primary analytic sample, we conducted a sensitivity analysis restricted to participants for whom the temporal sequence of baseline STQ assessment, UPPS negative urgency assessment, and nicotine use initiation was preserved. Of the 239 nicotine use initiators in the primary sample, 109 had their selected UPPS assessment before nicotine use onset, whereas 130 had recorded nicotine use onset before the selected UPPS assessment. Exclusion of the latter participants yielded 5835 participants and 109 events; after restriction to complete covariate data, the adjusted sensitivity models included 5345 participants and 96 events.

Weekend texting remained associated with subsequent nicotine use initiation in the temporally ordered sample (HR=1.11, 95% CI 1.06‐1.16; *P*<.001). After adjustment for negative urgency, the association remained significant (HR=1.10, 95% CI 1.05‐1.16; *P*<.001), and negative urgency was independently associated with nicotine use initiation (HR=1.11, 95% CI 1.02‐1.21; *P*=.01). Inclusion of negative urgency attenuated the weekend texting log-hazard coefficient by 6.8%. There was no evidence of violation of the proportional hazards assumption (global *P*=.83 for both models).

### Sensitivity Analysis Using Visit-Based Nicotine Use

Sensitivity analyses using a fallback definition of nicotine use initiation based on the first observed nicotine use at follow-up visits yielded consistent results with slightly larger effect sizes. In these models, both weekend texting screen time and negative urgency remained strongly associated with nicotine use initiation, and model discrimination was higher, reflecting increased event capture in this framework.

Overall, the association between weekend texting and nicotine use initiation was robust across alternative outcome definitions.

## Discussion

### Principal Findings

In this longitudinal analysis of nicotine-naive early adolescents, greater weekend texting screen time was associated with increased risk of subsequent nicotine use initiation. This association persisted after adjustment for demographic covariates and remained statistically significant after adjustment for negative urgency. Weekend texting was also positively associated with negative urgency, and negative urgency was independently associated with nicotine use initiation. Adjustment for negative urgency produced modest attenuation of the texting-initiation association, suggesting that the two measures capture partially overlapping information relevant to early nicotine use risk.

Importantly, negative urgency was not uniformly measured before nicotine use initiation in the primary analytic sample, precluding a strong temporal or causal mediation interpretation. However, in a sensitivity analysis restricted to participants with temporally ordered STQ, UPPS, and nicotine use onset assessments, both weekend texting and negative urgency remained associated with subsequent nicotine use initiation. These findings support the robustness of the observed associations while indicating that negative urgency accounts for only a modest portion of the texting-initiation relationship.

### Interpretation of Effect Sizes

Although the per-hour association was modest, larger exposure differences were associated with clinically meaningful increases in nicotine use initiation risk. A 5-hour increase in weekend texting corresponded to an approximately 30% to 40% higher hazard of initiation, with modest attenuation after accounting for negative urgency. Consistent with this, standardized models indicated that a 1-SD increase in weekend texting screen time was associated with a 21% higher hazard of initiation. Given the highly skewed distribution of texting behavior, such increments represent meaningful shifts in exposure and suggest that high-intensity digital engagement may carry substantive risk.

Negative urgency demonstrated a robust association with use initiation. A 1-SD increase in negative urgency corresponded to approximately 50% higher hazard of nicotine use onset, consistent with extensive literature linking urgency-related impulsivity to early substance use and escalation. These findings reinforce the central role of affect-driven impulsive behavior in adolescent nicotine vulnerability.

### Comparison With Existing Literature

Prior research has demonstrated associations between overall screen time and adolescent substance use, including smoking and vaping [8,9]. However, much of this literature has relied on cross-sectional designs, aggregated heterogeneous forms of screen behavior, or failed to establish temporal ordering between exposure and initiation [10,17]. By restricting analyses to nicotine-naive adolescents and using prospective time-to-event modeling, the present study strengthens inference regarding temporal sequencing.

Our focus on weekend texting should not be interpreted as evidence that weekend exposure is uniquely associated with nicotine use initiation or that weekday texting and other interactive digital behaviors are unimportant. Rather, weekend texting represents the specific measure of discretionary, socially interactive digital engagement examined in the present study. Future studies directly comparing weekday and weekend texting and other screen modalities will be necessary to determine whether the observed association is specific to weekend messaging or reflects broader patterns of digital social engagement.

Few studies have examined specific interactive forms of screen engagement such as texting and messaging. Text-based communication may differ from passive screen behaviors (eg, television viewing) in that it involves real-time social interaction, emotional salience, and peer reinforcement [15,28]. Consistent with this distinction, prior literature has suggested that screen time requiring interaction with others, such as texting, may be more strongly associated with vaping than more passive forms of screen use, such as watching videos [29]. Such environments may amplify social contagion effects or increase exposure to peer norms related to substance use [30,31].

The observed role of negative urgency aligns with theoretical models emphasizing affect regulation and impulsivity as key drivers of adolescent risk behavior. Youth high in negative urgency may be more likely to engage in maladaptive coping strategies, including substance use, particularly in emotionally charged contexts [11,12,14]. Digital communication environments may heighten emotional reactivity, social comparison, or interpersonal stress, thereby interacting with urgency-related traits [16,28]. Our findings provide empirical support for this conceptual framework.

### Negative Urgency and Potential Mechanisms

The attenuation of the texting coefficient after inclusion of negative urgency suggests that negative urgency statistically accounts for a modest portion of the association between weekend texting and nicotine use initiation. This interpretation was supported by the temporally ordered sensitivity analysis, in which both weekend texting and negative urgency remained associated with subsequent nicotine use initiation. However, these findings should not be interpreted as establishing negative urgency as a causal mediator or as indicating that texting episodes directly provoke nicotine use through acute negative affect. Rather, greater digital social engagement and negative urgency may represent correlated behavioral and individual-level characteristics associated with vulnerability to early nicotine use initiation [32,33].

This pattern suggests that additional mechanisms, as well as sources of residual confounding, likely contribute. These may include peer network exposure and social influence processes [30], digital advertising and product marketing [34,35], sensation-seeking tendencies not fully captured through negative urgency [36], and sleep disruption associated with high digital engagement [37]. Future work should examine these pathways directly, potentially incorporating peer network analyses, ecological momentary assessment, or digital trace data.

### Generalizability

The large and diverse ABCD cohort provides an important opportunity to examine risk factors for nicotine use initiation beginning in early adolescence. However, participants were approximately 10 years old at the baseline texting assessment, and nicotine use initiation during the observed follow-up therefore represents relatively early onset. The findings should consequently not be generalized to nicotine use initiation across the full adolescent age range, particularly initiation occurring during mid- or late adolescence, when prevalence is substantially higher. Instead, the present results identify behavioral and individual difference correlates of comparatively early nicotine use initiation. Replication across later developmental periods will be important. Because assessments used in the present analyses spanned 2019 to 2024, the observed associations reflect the nicotine product and digital media environments during that period; both environments have continued to evolve.

### Limitations

Several limitations warrant consideration. First, texting screen time was self-reported and may be subject to recall bias or social desirability effects. Objective digital use metrics would strengthen future studies. Second, although the prospective design improves temporal inference, residual confounding cannot be excluded. Unmeasured variables such as peer substance use, parental monitoring, or mental health symptoms may partially account for observed associations. Additionally, other screen modalities, particularly social media use, may confound this association as adolescents who text heavily on weekends may also have greater exposure to peer modeling or nicotine-related content online.

Third, the timing of the negative urgency assessment varied across participants. For approximately half of the analytic sample, the UPPS assessment took place within 1 day of the STQ assessment, whereas for others, the selected UPPS assessment took place substantially later. Moreover, among some participants who initiated nicotine use, the selected UPPS assessment took place after the recorded nicotine use onset date. This timing precludes interpretation of the primary negative urgency analyses as demonstrating causal mediation. We therefore interpret negative urgency as statistically accounting for part of the observed association rather than as an established causal intermediary. Reassuringly, sensitivity analyses restricted to participants for whom STQ assessment preceded UPPS assessment and UPPS assessment preceded nicotine use initiation yielded consistent associations for both weekend texting and negative urgency. Nevertheless, studies with prospectively scheduled and clearly separated exposure, mediator, and outcome assessments are needed to establish mediation.

### Future Directions

Future research should extend this work in several ways. First, incorporating objective digital use data would allow for more precise quantification of exposure patterns and timing. Second, examining additional impulsivity facets and affect regulation constructs may clarify which personality dimensions most strongly link digital engagement to substance use risk. Third, integrating peer network measures could test whether social contagion mediates part of the association. Fourth, longitudinal models that evaluate reciprocal influences among digital engagement, impulsivity development, and substance experimentation would deepen mechanistic understanding.

From a prevention standpoint, these findings suggest that adolescents with high digital engagement and elevated negative urgency may benefit from interventions targeting emotion regulation and coping skills. Digital literacy efforts may also help reduce exposure to substance-related content in online social environments.

### Conclusions

In a longitudinal cohort of nicotine-naive early adolescents, greater weekend texting screen time was associated with a higher hazard of subsequent nicotine use initiation. Negative urgency was independently associated with initiation and modestly attenuated the texting-initiation association. These associations persisted in a sensitivity analysis requiring temporal ordering of texting assessment, negative urgency assessment, and nicotine use initiation. Although these findings do not establish negative urgency as a causal mediator, they suggest that digital engagement and affect-driven impulsivity represent partially overlapping correlates of early nicotine use risk. Future longitudinal studies with clearly separated assessment of exposure, potential mediators, and nicotine use onset are needed to clarify the mechanisms underlying these associations.

## Supplementary material

10.2196/100303 Multimedia Appendix 1 Supplementary materials.

## Abbreviations

ABCD: Adolescent Brain Cognitive Development
HR: hazard ratio
IRB: institutional review board
STQ: Screen Time Questionnaire
UPPS: Urgency, Premeditation, Perseverance, and Sensation Seeking
