# Supplementary material for: Weekend Texting Screen Time and Incident Nicotine Use in Early Adolescents: Longitudinal Analysis of the Adolescent Brain Cognitive Development Study
Source: JMIR Pediatr Parent. 2026 Oct 13;9:e100303. doi: 10.2196/100303 (PMC13660968; doi:10.2196/100303)
Supplement: Supplementary file 1 [file pediatrics-v9-e100303-s001.docx]

**Supplementary Table 1: Fully Adjusted Cox Proportional Hazards Model for Time to Nicotine Initiation**

| **Variable** | **HR** | **95% CI** | **p-value** |
| --- | --- | --- | --- |
| *Weekend texting screen time (per hour)* | 1.06 | 1.03–1.10 | <0.001 |
| *Negative urgency (UPPS)* | 1.18 | 1.12–1.25 | <0.001 |
| *Female (vs male)* | 1.65 | 1.26–2.17 | <0.001 |
| *Age at baseline* | 1.31 | 1.07–1.61 | 0.010 |
| *Race: Black (vs White)* | 0.60 | 0.35–1.02 | 0.059 |
| *Race: Other/Multiracial (vs White)* | 0.77 | 0.53–1.13 | 0.184 |
| *Ethnicity: Hispanic/Latino (vs non-Hispanic)* | 1.13 | 0.78–1.64 | 0.522 |
| *Household income* | 0.97 | 0.91–1.05 | 0.474 |

Hazard ratios (HRs) and 95% confidence intervals (CIs) are shown for the association between baseline weekend texting screen time, negative urgency, and time to first nicotine use in adolescents who were nicotine-naïve at baseline. Time-to-event was defined as the number of days from the baseline assessment to the earliest recorded nicotine use onset, with participants censored at their last available follow-up. The model includes negative urgency to evaluate attenuation of the weekend texting association and adjusts for sex, age at baseline, race, ethnicity, and household income, with family-clustered robust standard errors. Reference categories are male sex, White race, and non-Hispanic ethnicity. Estimates correspond to the primary adjusted model reported in the main analyses
